# Environmental Pyrethroid Exposure and Cognitive Dysfunction in U.S. Older Adults: The NHANES 2001–2002

**DOI:** 10.3390/ijerph182212005

**Published:** 2021-11-16

**Authors:** Ui-Jin Kim, Myeongjin Hong, Yoon-Hyeong Choi

**Affiliations:** 1 Department of Occupational and Environmental Medicine, Gachon University Gil Medical Center, Incheon 21565, Korea; ujkim@gilhospital.com; 2 Department of Preventive Medicine, Graduate School of Medicine, Gachon University, Incheon 21999, Korea; a201806@gmail.com; 3 Department of Health Sciences and Technology, GAIHST, Gachon University, Incheon 21999, Korea

**Keywords:** cognition, pyrethroid, pesticide, older adults, NHANES

## Abstract

Pyrethroid compounds are widely used in household insecticides and agricultural pesticides. Recent studies, however, report that pyrethroid exposures affect neurobehavioral function in animals and may be associated with adverse neurocognitive development in children. This study aimed to examine the association between pyrethroid exposure and cognitive dysfunction in older adults using a well-defined general population. We analyzed data from 336 individuals, aged 60–84 years, who participated in the National Health and Nutrition Examination Survey 2001–2002. We used urinary 3-phenoxybenzoic acid (3-PBA) concentration as a biomarker of pyrethroid exposures and assessed cognitive function with the digit–symbol coding test. The geometric means (±geometric standard errors) of creatinine-uncorrected and corrected urinary 3-PBA were 0.30 (±0.87) μg/L and 0.36 (±0.89) μg/g. After adjusting for sociodemographic factors, higher 3-PBA concentrations (> vs. ≤0.30 μg/g creatinine (median)) were associated with lower scores of cognitive function (−3.83 95% confidence interval: −7.11, −0.54). Significance was persistent after additionally adjusting for physical activity and smoking pack-year (−3.76 95% CI: −7.16, −0.36) and further adjusting for BMI and presence of hypertension and diabetes (−3.82 95% CI: −6.92, −0.71). Our findings suggest that pyrethroid exposure is associated with cognitive dysfunction in older adults.

## 1. Introduction

Pyrethroids are analogs of natural pyrethrins produced by pyrethrums and are widely used in household insecticides and agricultural pesticides [1]. The utility of pyrethroids is based on their disruption of gating of insect axonal sodium channels so that neurons are damaged due to hyperstimulation [2]. When pyrethroids enter a human body, they are rapidly metabolized via several pathways [3], one of which is their hydrolysis to 3-phenoxybenzyl alcohol or 3-phenoxybenzaldehyde, a metabolite that is rapidly transformed to 3-phenoxybenzoic acid (3-PBA).

Although pyrethroid insecticides have commonly been considered to be safer than other insecticides such as organochlorine, organophosphorus, and methylcarbamates [4], there is growing experimental evidence of pyrethroid’s neurotoxicity [5]. Recent epidemiological studies show that low-level pyrethroid pesticide exposures are associated with neurobehavioral cognitive dysfunction in children. One study of Nicaraguan children reports that organophosphate and pyrethroid pesticide exposures adversely affect neurobehavioral development [6]. Another study of a mother–child cohort reports that childhood exposures to pyrethroid were negatively associated with neurocognitive development, particularly in verbal comprehension and working memory [7]. Despite current epidemiological studies on pyrethroid and neurobehavioral cognitive development in children, there are few studies on pyrethroid and neurobehavioral cognitive impairment in older adults. In general, environmental toxicity on poor neurocognitive outcome may affect the development in childhood as well as the degeneration in adulthood. A review study reported that heavy metals, air pollution, and organic compounds are known to delay cognitive development in child, and also could affect declines in cognition in adults and cause neurodegenerative diseases such as dementia and Alzheimer’s disease [8]. Indeed, experimental studies of adult animals report that acute oral exposure to low-dose pyrethroids impair schedule-controlled operant responding, decreased grip strength, and produced incoordination in adult rats [9,10,11], while there have been still unknown for cognitive impairment. 

According to the World Health Organization, the number of people with dementia in the world is estimated at 47.5 million in 2015, and is predicted to increase to 75.6 million by 2030 and to more than triple by 2050. In general, dementia, or low cognitive function, is linked to poor quality of life [12]. Because dementia is dependent on elevated age [13,14] and the human lifespan is continuously increasing, the number of people vulnerable to dementia is growing and it is important to investigate risk factors for dementia. 

In the present study, we therefore examined the cross-sectional association between urinary 3-PBA concentrations and cognitive function in older adults from the National Health and Nutrition Examination Survey (NHANES) 2001–2002.

## 2. Materials and Methods

### 2.1. Study Population

The NHANES is a cross-sectional and population-based survey to obtain health and nutrition status conducted by the National Center for Health Statistics (NCHS) of the Centers for Disease Control and Prevention (CDC). The NHANES is constructed in a stratified, clustered design and provides demographic, socioeconomic, behavioral, and dietary data of a representative sample of the U.S. general population. Each survey participant provided demographic and health history information through household surveys, and blood and urine samples and physical examinations were taken at a mobile examination center (MEC) [15].

We used data from the NHANES survey of 2001–2002, the only years that include data of both urinary 3-PBA concentration and cognitive function. Since subjects aged 60 years or more were assigned to take the cognitive function test, and NHANES subjects aged 85 years or more were coded as 85 years (their exact age is not available), the current study included participants between 60 and 84 years of age. Hence, our statistical analysis includes only those 336 participants aged 60–84 years who have information on urinary 3-PBA concentration, cognitive function, and covariates. 

### 2.2. Urinary Pyrethroid Metabolite Measurement

The common metabolite, 3-PBA, contains synthetic pyrethroids including permethrin, cypermethrin, deltamethrin, allethrin, resmethrin, fenvalerate, cyhalothrin, fenpropathrin, and tralomethrin [16] and has a half-life of about 6–24 h [17,18,19,20]. We used urinary 3-PBA concentration as a biomarker of environmental pyrethroid exposure. At the MEC, urine samples were collected from every participant aged 6 or above, and were stored frozen at −20 °C until being transported to the National Center for Environmental Health laboratory [21]. Urinary 3-PBA concentrations were measured on a randomly selected one-third subsample of the NHANES participants. The samples were analyzed according to the following method [22]. Stable isotopically labeled analogues of 3-PBA were mixed with urine as an internal standard. The hydrolysates were prepared through the solid phase extraction procedure (OASIS HLB extraction cartridge plate (waters Corp., Milford, MA, USA)) and were analyzed by liquid chromatography with tandem mass spectroscopy (LC/MS/MS: Agilent 1100 Series LC system (Agilent Technologies, Santa Clara, CA, USA); ThermoFisher TSQ Quantum Ultra triple-quadrupole mass spectrometer (ThermoFisher Scientific Inc., Waltham, MA, USA)). The limit of detection (LOD) is 0.1 µg/L, and urinary 3-PBA concentrations below the LOD were assigned as the LOD divided by the square root of two (*n* = 110) [21].

### 2.3. Cognitive Function

The NHANES uses the Wechsler Adult Intelligence Scale III Digit-Symbol Coding Test (DSCT) to examine cognitive function in older adults. The DSCT is commonly used to measure brain damage, dementia, memory, attention, and motor ability in adults aged 60 years or more [23,24]. During their examination, participants draw symbols corresponding to the numbers provided at the top of the examination paper. Each correct symbol has 1 point score, with a total possible score of 133 (higher score indicates greater cognitive function). Participants have only the time limit of 120 seconds to respond to each question [25].

### 2.4. Covariates

There are several potential confounding factors to be considered in examining associations between urinary 3-PBA and cognitive function. First, demographic factors including age, sex, and race/ethnicity were considered. Race/ethnicity was classed as non-Hispanic white, non-Hispanic black, Mexican American, and other races. Second, socioeconomic status including educational level, and income were considered. Educational level was classed as high school diploma, less than high school diploma, and more than high school diploma. Household income was divided into 2 groups based on the poverty-income ratio (PIR): PIR < 1 and PIR ≥ 1. Third, general health behaviors including physical activity and smoking pack-year were additionally considered. Last, physical status including body mass index (BMI) and presence of hypertension and diabetes were additionally considered as confounding variables [26,27]. Further, urinary creatinine concentration was considered as a potential confounder [28]. These confounding factors were selected based on previous studies [29,30].

### 2.5. Statistical Analysis

To account for the complex survey design and sample weights of NHANES, we used the SAS 9.4 (SAS institute, Inc., Cary, NC, USA) survey procedure for all analyses. We used two-year sample weights that were adjusted for oversampling of certain subpopulation and non-response according to the NCHS recommendation [31]. The independent variable, urinary 3-PBA concentration, was right skewed and natural log-transformed to normalize. Geometric means of 3-PBA were computed, and their geometric means by participant characteristics were compared, using survey t-test for binominal groups and Wald F-test for categorical group. The dependent variable, cognitive function described by the DSCT, was used directly in the regression models since it follows a normal distribution. In order to examine the associations between urinary 3-PBA concentrations and DSCT scores, we used a multivariable linear regression model (SURVEYREG). Because of non-linearity between urinary 3-PBA and the loss of cognitive function in a small study population, we modeled urinary 3-PBA as a binominal group (cut-point: median), and compared groups of upper vs. lower 3-PBA concentrations (note that we simply compared the difference of cognitive function in the two groups of high and low urinary 3-PBA levels, since sample size is not large.). Confounding factors were considered in the multivariable regression models in four different ways: Model (A) age, sex, race/ethnicity, and urinary creatinine level; Model (B) educational level, income, and the all confounding factors considered in the model A; Model (C) physical activity, smoking pack-year, and all confounding factors considered in Model (B); Model (D) BMI, hypertension, diabetes, and all confounding factors considered in Model (C). To account for urine dilution, 3-PBA concentrations were either adjusted or divided by urine creatinine (µg/g).

## 3. Results

Table 1 shows participant characteristics of 336 adults between 60 and 84 years of age. The weighted mean (±standard error (SE)) of age was 70.5 (±0.3) years; 44.5% of participants were males. Overall, mean of the cognitive function score (±SE) using DSCT was 48.4 (±1.5). Among levels of race/ethnicity, the largest group was non-Hispanic whites (84.8%); the largest group in the educational level were people with higher than high school diploma (44.0%); 92.3% of study participants were at income levels above poverty; 71.7% of all study participants had hypertension and 14.1% of all study participants had diabetes. 

Table 2 shows geometric means of urinary 3-PBA concentrations by participant characteristics. The geometric means (±geometric SE) of creatinine-uncorrected and corrected urinary 3-PBA were 0.30 (±0.04) μg/L and 0.36 (±0.04) μg/g. Creatinine-uncorrected urinary 3-PBA varied significantly by race/ethnicity (*p* = 0.027). Participants with higher urinary creatinine (≥91.0 mg/dL) had significantly higher urinary 3-PBA than those with lower urinary creatinine level (*p* < 0.001). The distribution of creatinine-corrected urinary 3-PBA was different from that of creatinine-uncorrected urinary 3-PBA. Females had significantly higher urinary levels of 3-PBA than did males (*p* = 0.013), and urinary 3-PBA varied by cumulative cigarette pack-year (*p* = 0.031). Urinary 3-PBA concentration did not significantly vary by all other variables not referred to here.

Table 3 shows associations between cognitive function and urinary 3-PBA, analyzed according to the multivariable linear regression models. In creatinine-corrected 3-PBA models, the cognitive function score in high 3-PBA group (>0.30 μg/g (median)) was not significantly lower than that in low 3-PBA group when adjusted for age, sex and race/ethnicity (Model A). After additional adjustment for education and PIR, changes in the cognitive function score became significantly lower in high 3-PBA group (Model B; −3.83 95% CI: −7.11, −0.54). The significance remained after additional adjustment for physical activity and smoking pack-year (Model C; −3.76 95% CI: −7.16, −0.36) as well as with further adjustment for BMI and presence of hypertension and diabetes (Model D; −3.82 95% CI: −6.92, −0.71). No significant change in cognitive function score was observed in all models when we performed urinary 3-PBA concentrations after adjusting for creatinine as a confounder (instead of calculating 3-PBA concentrations divided by urine creatinine). Results that modeled 3-PBA concentrations as tertiles were shown in Supplementary Table S1.

## 4. Discussion

In the present study of U.S. older adults aged 60–84 years, higher exposure to pyrethroid compounds, as estimated by urinary 3-PBA concentrations, was associated with lower scores of cognitive function. Our observations were made after adjusting for important potential confounders: age, sex, race/ethnicity, education level, PIR, physical activity, smoking pack-year, BMI, and the presence of hypertension and diabetes. There are few previous examinations of the effect of pyrethroid exposure on cognition among adults, although several studies have reported effects of pyrethroid exposure on cognition among children.

Indeed, one series of investigations by Richardson et al. provides evidence for attention deficit through both animal and human children studies. Their study reports that mice that were exposed to deltamethrin, a pyrethroid compound, during development showed several features of attention deficit hyperactivity disorder (ADHD), that is, elevated dopamine transporter level, hyperactivity, working memory and attention deficits, and impulsivity. They explain that increased dopamine transporter (DAT) and D1 dopamine receptor levels may be responsible for these behavioral deficits. In their human studies, nested case-control reports that children aged 6–15 years with detectable urinary pyrethroid metabolites were twice as likely to have ADHD compared to those with no such metabolites [32]. In addition, another study using children of Nicaraguan agricultural workers reported that pyrethroid exposure during the first year of life may reduce perceptual reasoning function in children aged 7–9 years [33]. 

However, there has been limited study of adults’ pyrethroid exposure. To our knowledge, only one crude report has examined the association of pyrethroid exposure with cognitive dysfunction in the U.S. adults, but it failed to observe their significant associations. That study modelled the digit symbol substitution test (DSST) score as a dichotomous variable (good vs. poor cognition) and urinary pyrethroid concentrations as quartiles [26]. The current study deals with DSST score as a continuous variable (in order to capture slight change in cognition) as well as urinary pyrethroid as binominal (due to excessive number of subjects below detection rate). Kim et al.’s study may observe null findings because pyrethroid exposure in the general population might not be at levels high enough to induce cognitive dysfunction as defined by threshold. 

Whereas previous studies on pyrethroid exposure have provided evidence on its neurotoxicity in mammals, the mechanism of pyrethroid’s effect in decreased cognitive function has not been clarified. Pyrethroids stimulate neurons excessively, resulting in neuronal damage. It is well known that excitotoxins can cause acute symptoms of poisoning and chronic neuronal degenerative change [34], and are associated with various neurodegenerative diseases such as multiple sclerosis, Alzheimer’s disease, and Parkinson’s disease [35]. The cumulative excitotoxic effect of pyrethroid may thereby potentially explain our observations in the associations between pyrethroid exposure and cognitive dysfunction in adults. 

Since the 1970s, pyrethroids have been replacing organophosphates and other insecticide that are known to be toxic to mammals and so have been more widely used in household insecticides and agricultural pesticides [16,36]. Data on the human health effects of pyrethroids encountered by the public are currently limited. However, there is updated evidence indicating that even at low environmental doses, pyrethroid exposure may have adverse effects in other health outcomes [37]. Our findings provide further evidence supporting concern for current levels of exposure to pyrethroid. 

In the present study, we used urinary 3-PBA concentration as a biomarker of pyrethroid exposures it is a metabolite of up synthetic pyrethroids such as permethrin, cypermethrin, deltamethrin, allethrin, resmethrin, fenvalerate, cyhalothrin, fenpropathrin, and tralomethrin. Although the NHANES 2001–2002 has data on other urinary metabolites of pyrethroid compounds, e.g., urinary cis- and trans-3-(2,2-dichlorovinyl)-2,2-dimethylcyclopropane carboxylic acid (cis- and trans-DCCA), 4-fluoro-3-PBA (4-F-3-PBA), and cis-3-(2,2-dibromovinyl)-2,2-dimethylcyclopropane carboxylic acid (cis-DBCA), we were not able to analyze their associations because the detection rates for these metabolites were too low for reliable statistical analysis.

This study has several strengths. We used data from the NHANES, a representative and generalizable sample of the U.S. population. In addition, we controlled various important potential confounders in statistical analyses. Thus, the results of this study provide reliable information about the relationship between pyrethroid exposure and cognitive dysfunction.

However, several limitations exist in this study. First, this study is cross-sectional, so we cannot rule out the possibility of an inverse causal relation. Second, urinary 3-PBA has an estimated half-life of only 6–24 h [17,18,19,20], and thus may not be able to capture cumulative pyrethroid exposure in the human body. Third, we adjusted clinical factors such as BMI, hypertension, and diabetes, as potential confounders, as previous studies suggested [26,27]. However, we cannot rule out a possibility that those clinical factors may be potential causal intermediaries rather than confounding factors since they are the risk factor for cognitive impairment in older adults. Fourth, there may be residual confounding including metabolizer types, despite the adjustment for various potential confounding factors. Fifth, since sample size is not large, we simply compared the difference of cognitive function in the two groups of high and low urinary 3-PBA levels. Thus, we were not able to examine dose-dependent effects following the various urinary 3-PBA changes. Future studies using a larger and longitudinal population are needed to confirm our findings for associations between pyrethroid exposure and cognitive dysfunction. 

## 5. Conclusions

Our findings from a representative sample of the U.S. general population suggest that pyrethroids may be associated with cognitive dysfunction in older adults. Because pyrethroid chemicals have been used worldwide in recent decades, strong efforts to regulate and reduce environmental levels of pyrethroid exposure are required in order to prevent chemically-induced cognitive dysfunction and its related decrease in quality of life for older adults.

## Figures and Tables

**Table 1:** Participant characteristics (*n* = 336 ^1^).

Characteristic	All Participants
Age (years) ^2^	70.5 (±0.3)
Urinary 3-PBA concentration (µg/L) ^3^	0.30 (±0.04)
Cognitive function score ^2^	48.4 (±1.5)
Urinary creatinine concentration (mg/dL) ^2^	102.6 (±3.5)
BMI (kg/m^2^)	28.4 (±0.3)
Sex (%)	
Male	44.5
Female	55.5
Race/ethnicity (%)	
Non-Hispanic white	84.8
Non-Hispanic black	6.4
Mexican American	2.4
Other	6.4
Education (%)	
<High school	27.5
High school diploma	28.5
>High school	44.0
Income (%)	
Below poverty (<1)	7.7
Above poverty (≥1)	92.3
Moderate or vigorous physical activity (%)	
Yes	80.3
No	19.7
Hypertension (%)	
Yes	71.7
No	28.3
Diabetes (%)	
Yes	14.1
No	85.9

^1^ Participants (*n =* 336) are the individuals having all interest variables in this study: age, urinary 3-PBA, cognitive function score, urinary creatinine, BMI, sex, race/ethnicity, education, income, physical activity, hypertension and diabetes. ^2^ Arithmetic mean (arithmetic standard error) was presented. ^3^ Geometric mean (geometric standard error) was presented. 3-PBA, 3-phenoxybenzoic acid; BMI, body mass index.

**Table 2:** Geometric means and geometric standard errors of urinary 3-PBA concentrations by participant characteristic (*n =* 336).

Characteristic	Participants *n* (%) ^1^	Urinary 3-PBA Uncorrected (μg/L)	*p*-Value ^2^	Urinary 3-PBA Creatinine-Corrected (μg/g)	*p*-Value ^2^
Total	336	0.30 (±0.04)		0.36 (±0.04)	
Age (years)					
60–69	147 (48.2)	0.31 (±0.06)		0.38 (±0.06)	
70–79	113 (35.2)	0.27 (±0.04)		0.32 (±0.04)	
80–85	76 (16.6)	0.32 (±0.09)	0.833	0.39 (±0.10)	0.641
Sex					
Male	158 (44.5)	0.30 (±0.04)		0.28 (±0.03)	
Female	178 (55.5)	0.30 (±0.05)	0.989	0.44 (±0.07)	0.013
Race/ethnicity					
Non-Hispanic white	228 (84.8)	0.28 (±0.04)		0.35 (±0.04)	
Non-Hispanic black	41 (6.4)	0.59 (±0.12)		0.56 (±0.12)	
Mexican American	49 (2.4)	0.21 (±0.06)		0.30 (±0.06)	
Other	18 (6.4)	0.40 (±0.24)	0.027	0.44 (±0.23)	0.185
Urinary creatinine (mg/dL)					
<91.0 ^3^	167 (47.5)	0.17 (±0.02)		0.37 (±0.05)	
≥91.0	169 (52.5)	0.50 (±0.07)	<0.001	0.35 (±0.05)	0.742
Education					
<High school	119 (27.5)	0.29 (±0.08)		0.33 (±0.08)	
High school	76 (28.5)	0.26 (±0.05)		0.36 (±0.07)	
>High school	141 (44.0)	0.34 (±0.06)	0.548	0.38 (±0.05)	0.879
Income					
Below poverty (<1)	31 (7.7)	0.28 (±0.21)		0.46 (±0.29)	
Above poverty (≥1)	305 (92.2)	0.30 (±0.03)	0.929	0.35 (±0.03)	0.689
Moderate or vigorous physical activity					
Yes	264 (80.3)	0.30 (±0.03)		0.37 (±0.04)	
No	72 (19.7)	0.30 (±0.10)	0.990	0.32 (±0.09)	0.657
Cumulative cigarette pack-years					
Never	164 (48.9)	0.35 (±0.05)		0.42 (±0.06)	
<20	72 (19.2)	0.33 (±0.08)		0.42 (±0.10)	
≥20	100 (31.9)	0.23 (±0.03)	0.061	0.26 (±0.02)	0.031
BMI (kg/m^2^)					
<30	234 (67.2)	0.29 (±0.05)		0.37 (±0.05)	
≥30	102 (32.8)	0.33 (±0.06)	0.580	0.34 (±0.06)	0.605
Hypertension					
Yes	240 (71.7)	0.31 (±0.05)		0.38 (±0.05)	
No	96 (28.3)	0.28 (±0.03)	0.542	0.32 (±0.04)	0.371
Diabetes					
Yes	49 (14.1)	0.32 (±0.04)		0.34 (±0.07)	
No	287 (85.9)	0.30 (±0.07)	0.790	0.36 (±0.05)	0.771

^1^ Weighted percentages from survey frequency. ^2^ Survey *t*-test for binominal groups and the Wald F-test for race/ethnicity and cumulative cigarette pack-years. ^3^ Cut-off point: median for urinary creatinine. 3-PBA, 3-phenoxybenzoic acid; BMI, body mass index.

**Table 3:** Difference (95% confidence interval) in cognitive function score between Cr-uncorrected 3-PBA and Cr-corrected 3-PBA (*n =* 336).

	Cognitive Function Score			
	Model A ^1^	Model B ^2^	Model C ^3^	Model D ^4^
Cr-uncorrected 3-PBA (μg/dL)				
Low (0.07~0.22)	0 (Reference)	0 (Reference)	0 (Reference)	0 (Reference)
High (0.23~47.94)	−1.09 (−5.60, 3.42)	−2.87 (−6.09, 0.36)	−2.72 (−6.06, 0.61)	−2.72 (−5.94, 0.50)
Cr-corrected 3-PBA (μg/g)				
Low (0.03~0.30)	0 (Reference)	0 (Reference)	0 (Reference)	0 (Reference)
High (0.30~42.52)	−3.24 (−7.51, 1.02)	−3.83 (−7.11, −0.54)	−3.76 (−7.16, −0.36)	−3.82 (−6.92, −0.71)

^1^ Model A was adjusted for age, sex, race/ethnicity (and creatinine for Cr-uncorrected 3-PBA model). ^2^ Model B was adjusted for all variables in Model A and further adjusted for education and PIR. ^3^ Model C was adjusted for all variables in Model B and further adjusted for physical activity and smoking pack year. ^4^ Model D was adjusted for all variables in Model C and further adjusted for BMI, hypertension and diabetes. Cr, creatinine; 3-PBA, 3-phenoxybenzoic acid; PIR, poverty-income ratio; BMI, body mass index.

## Data Availability

This analysis utilizes publicly available data, which can be downloaded from https://www.cdc.gov/nchs/nhanes/ (accessed on 23 September 2021).

## Supplementary Materials

The following are available online at https://www.mdpi.com/article/10.3390/ijerph182212005/s1, Table S1: Difference (95% confidence interval) of the cognitive score with trichotomous Cr-uncorrected 3-PBA and Cr-corrected 3-PBA (*n* = 336).
