# Supplementary material for: Environmental Pyrethroid Exposure and Cognitive Dysfunction in U.S. Older Adults: The NHANES 2001–2002
Source: Int J Environ Res Public Health. 2021 Nov 16;18(22):12005. doi: 10.3390/ijerph182212005 (PMC8623149; doi:10.3390/ijerph182212005)
Supplement: Supplementary file 1 [file ijerph-18-12005-s001.zip › ijerph-1413334-supplementary.pdf]

Supplementary Materials

Supplementary Table S1. Difference (95 % confidence interval) of the cognitive score with trichotomous Cr-uncorrected 3-PBA and Cr-corrected 3-PBA (*n* = 336)

	**Cognitive function score**			
	Model A[1]	Model B[2]	Model C[3]	Model D[4]
Cr-uncorrected 3-PBA (μg/dL)				
Tertile 1	0 (Reference)	0 (Reference)	0 (Reference)	0 (Reference)
Tertile 2	-0.39 (-7.21, 6.44)	-0.85 (-6.81, 5.10)	-0.32 (-6.36, 5.72)	-0.58 (-6.37, 5.21)
Tertile 3	-0.42 (-7.31, 6.47)	-1.68 (-7.30, 3.93)	-1.34 (-7.12, 4.44)	-1.56 (-7.01, 3.90)
p for trend	0.8987	0.5237	0.6106	0.5319
Cr-corrected 3-PBA (μg/g)				
Tertile 1	0 (Reference)	0 (Reference)	0 (Reference)	0 (Reference)
Tertile 2	0.90 (-2.33, 4.14)	0.58 (-2.48, 3.64)	0.54 (-2.71, 3.78)	0.47 (-3.08, 4.02)
Tertile 3	-0.24 (-5.71, 5.24)	-1.70 (-6.03, 2.63)	-1.70 (-6.44, 3.04)	-1.71 (-6.26, 2.85)
p for trend	0.9076	0.3958	0.4366	0.4167

[1] Model A was adjusted for age, sex, race/ethnicity (and creatinine for Cr-uncorrected 3-PBA model).
[2] Model B was adjusted for all variables in model A and further adjusted for education and PIR.
[3] Model C was adjusted for all variables in model B and further adjusted for physical activity and smoking pack year.
[4] Model D was adjusted for all variables in model C and further adjusted for BMI, hypertension and diabetes.
Cr, creatinine; 3-PBA, 3-phenoxybenzoic acid; PIR, poverty-income ratio; BMI, body mass index
